# Supplementary figures and images for: Convolutional Neural Network Transformer (CNNT) for Fluorescence Microscopy image Denoising with Improved Generalization and Fast Adaptation
Source: ArXiv. 2024 Apr 6:arXiv:2404.04726v1. Preprint. [Version 1] (PMC11188127)

Supplement Video 1

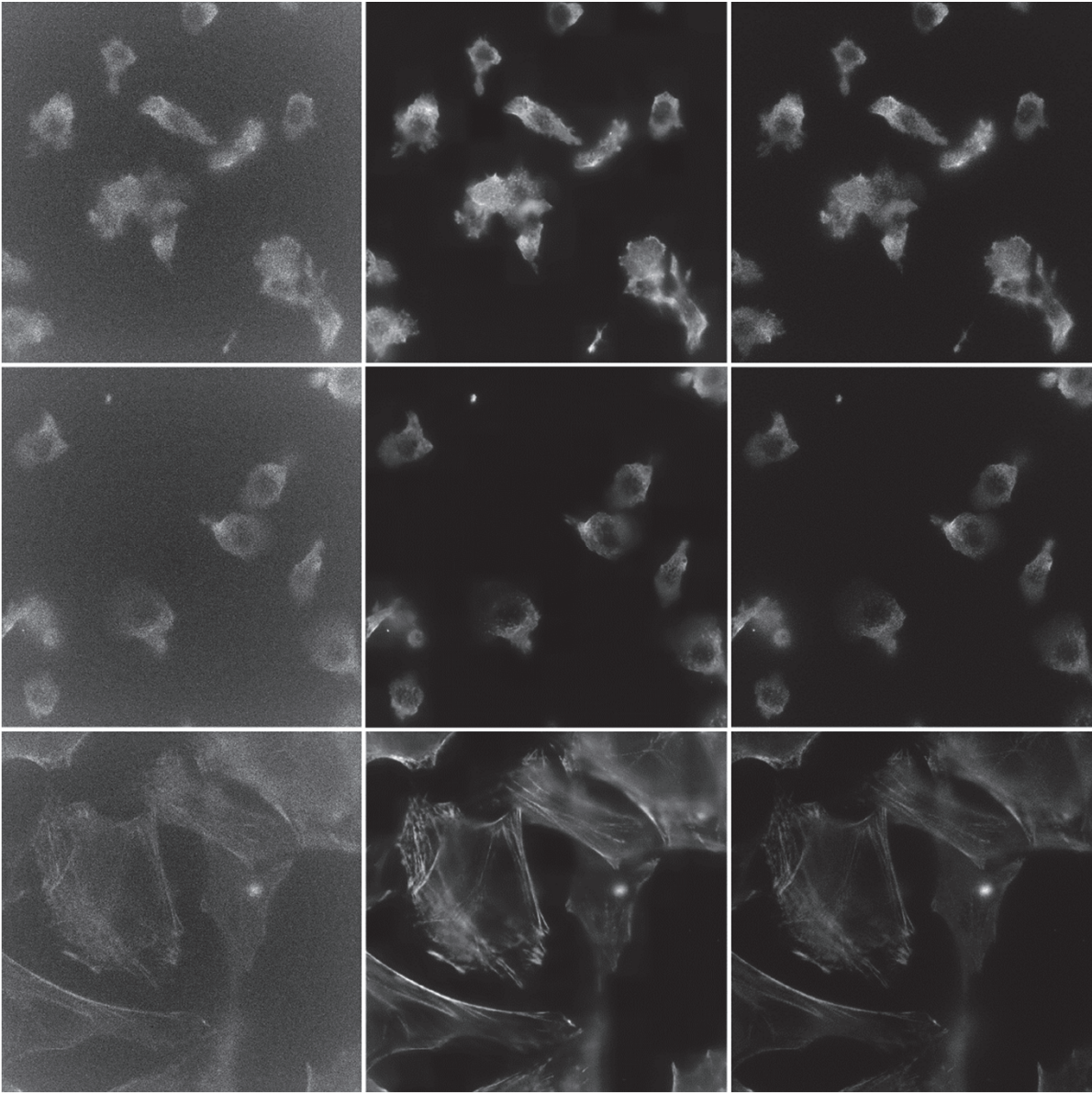

Supplement Video 2

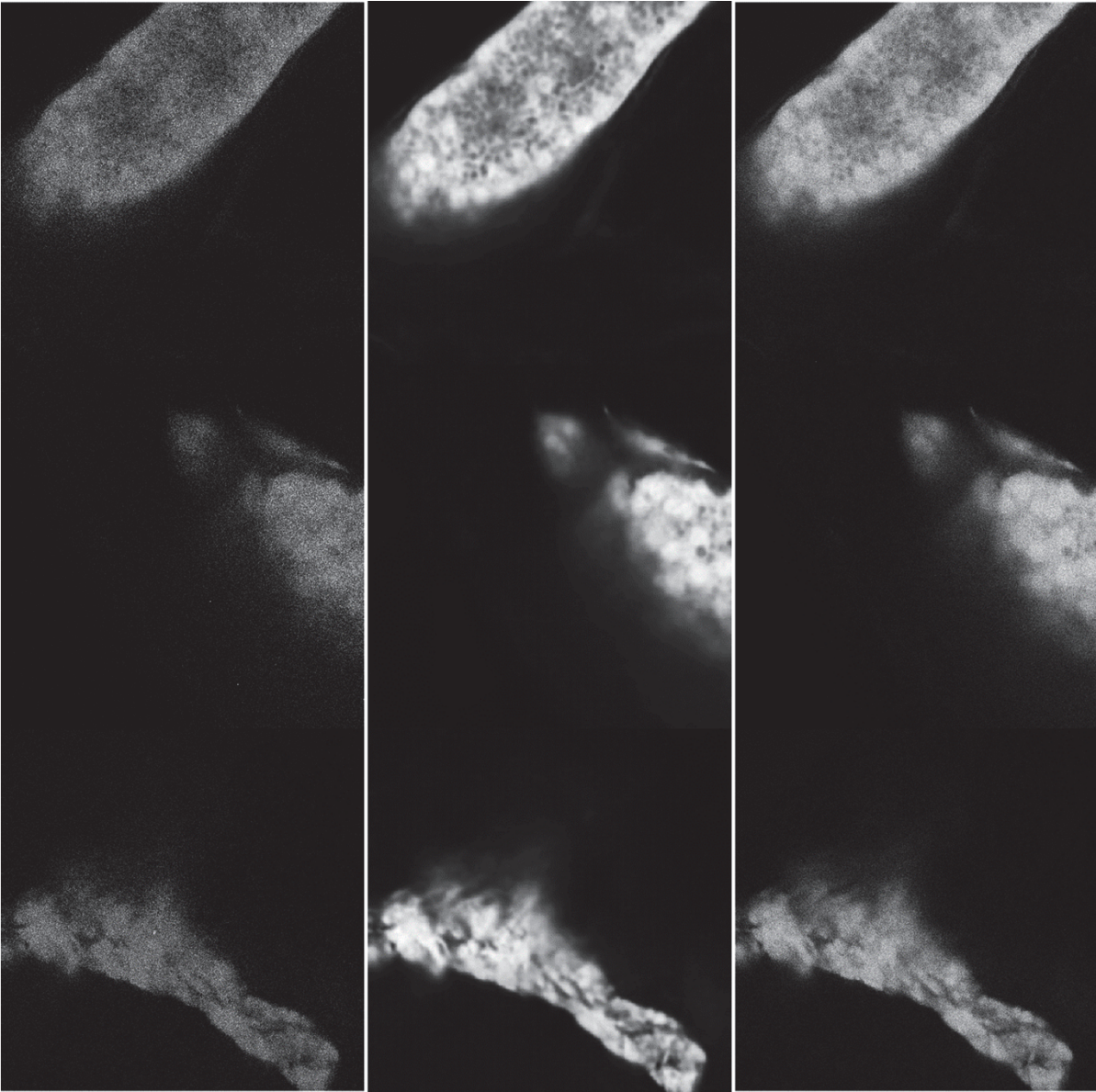

Supplement Video 3

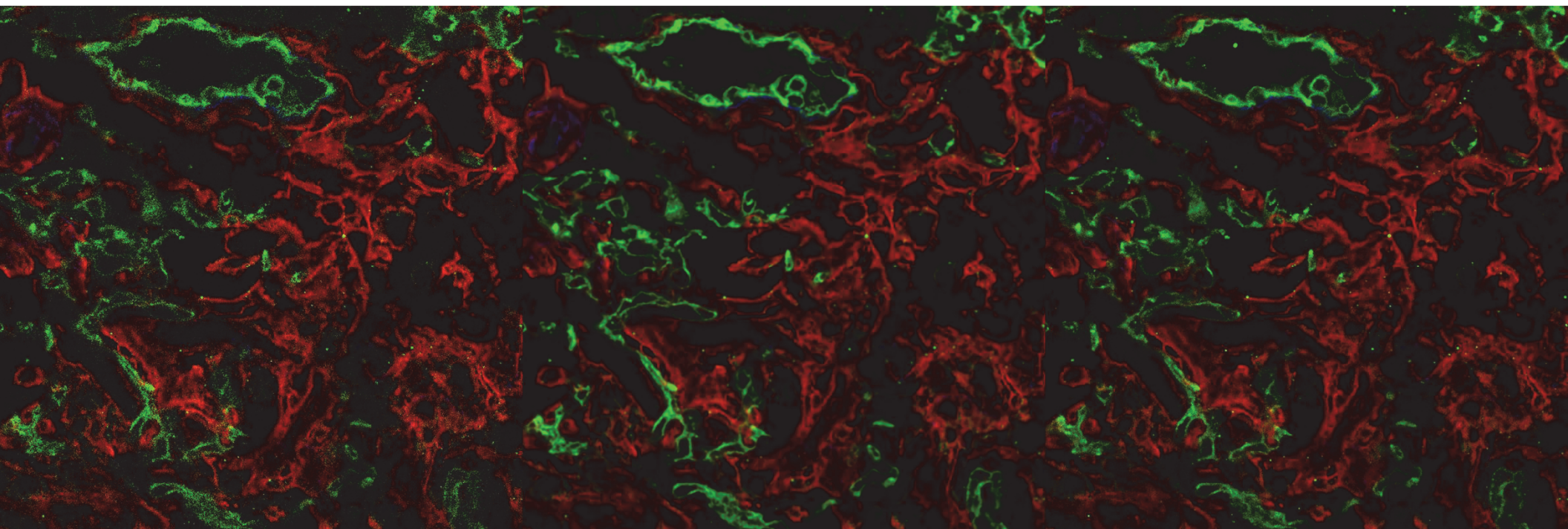

Supplement: Supplement 1 [file NIHPP2404.04726v1-supplement-1.pdf]
